# Sexual Dimorphism and Gender in Infectious Diseases

**DOI:** 10.3389/fimmu.2021.698121

**Published:** 2021-07-22

**Authors:** Laetitia Gay, Cléa Melenotte, Ines Lakbar, Soraya Mezouar, Christian Devaux, Didier Raoult, Marc-Karim Bendiane, Marc Leone, Jean-Louis Mège

**Affiliations:** ^1^Aix-Marseille Univ, IRD, APHM, MEPHI, IHU-Méditerranée Infection, Marseille, France; ^2^Aix-Marseille Univ, INSERM, IRD, SESSTIM, Economy and Social Science, Health Care Systems and Societies, Marseille, France; ^3^Department of Anaesthesia and Intensive Care, Hôpital Nord, Aix-Marseille Univ, APHM, Marseille, France

**Keywords:** sexual dimorphism, gender, infectious disease, sex hormones, personalized medicine

## Abstract

Epidemiological studies and clinical observations show evidence of sexual dimorphism in infectious diseases. Women are at less risk than men when it comes to developing most infectious diseases. However, understanding these observations requires a gender approach that takes into account an analysis of both biological and social factors. The host’s response to infection differs in males and females because sex differences have an impact on hormonal and chromosomal control of immunity. Estradiol appears to confer protective immunity, while progesterone and testosterone suppress anti-infectious responses. In addition, genetic factors, including those associated with sex chromosomes, also affect susceptibility to infections. Finally, differences in occupational activities, lifestyle, and comorbidities play major roles in exposure to pathogens and management of diseases. Hence, considering sexual dimorphism as a critical variable for infectious diseases should be one of the steps taken toward developing personalized therapeutic approaches.

## Introduction

**Figure 1:**
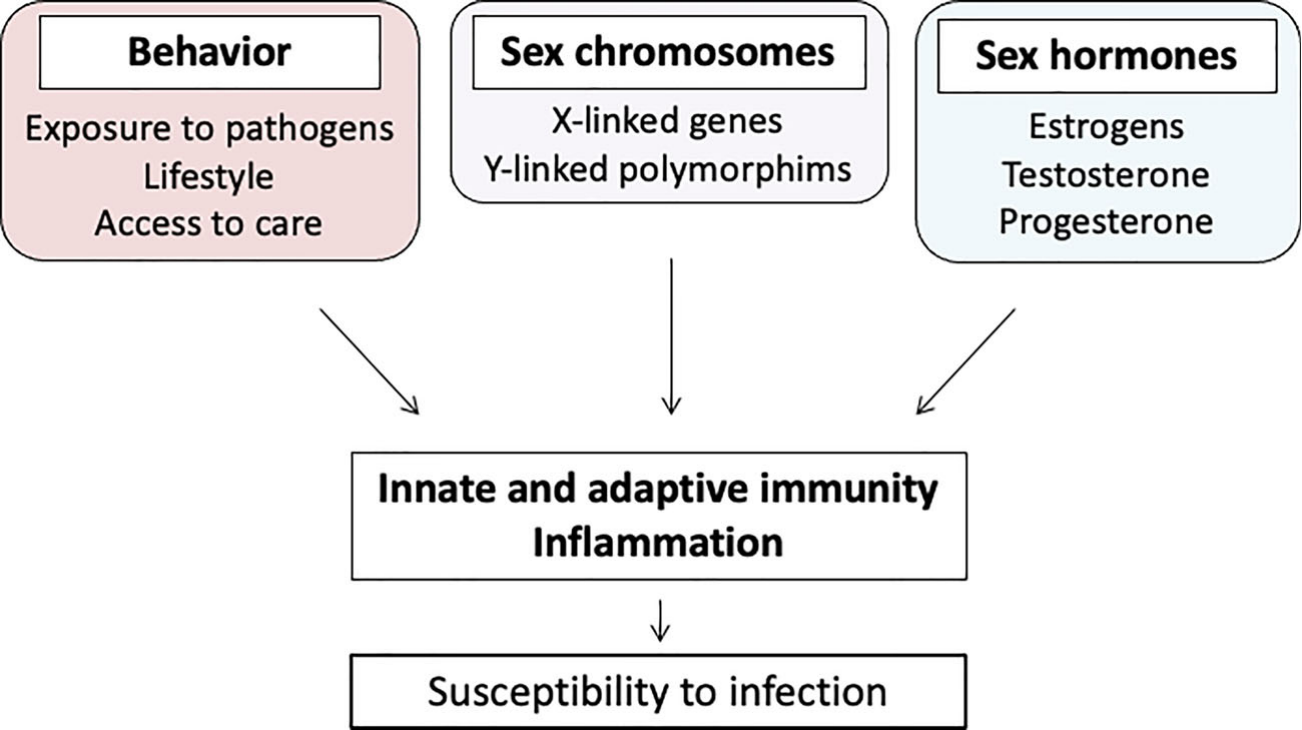
Multifactorial mechanisms of gender dimorphism.

In infectious diseases, the health differences between men and women are a result of interactions between biological and sociocultural factors. Hence, age, comorbidities, genetic predispositions, geographical distribution of pathogens, health behaviors, and hormonal influences are just some of the examples of the diversity of mechanisms explaining sex differences (1). These different factors make it difficult to discriminate what is related to sex as a biological entity and gender as a social construct. Indeed, comorbidities, exposure to pathogens, health behaviors, and access to healthcare interfere with the development of most infectious diseases (**Figure 1**) (2). Sexual dimorphism also affects the processes, including the immune system, used by the host to fight against infection (3, 4).

In infectious diseases, animal models and epidemiological studies provide strong support for sexual dimorphism, whereas mechanistic studies in patients are less conclusive. One reason for this dissociation is that biological studies are too reductionist, do not integrate sociocultural factors, and involve patients of each sex who have already developed the disease.

The recent events of the COVID-19 pandemic show the importance of sex differences in susceptibility to viral infections. This review is dedicated to updating the role of sexual dimorphism and gender differences in infectious diseases, here with the goal of providing a mechanistic approach toward infectious diseases.

### Search Strategy and Selection Criteria

For the purpose of the current review, we conducted a non-systematic search in Medline (through PubMed) and Google Scholar databases using the following keywords: “sex”, “gender”, “infection”, “bacteria”, “viruses”, “parasites”, “men”, “women”, “ratio”, and “difference”. The search was conducted with no restrictions on language or type of study, from inception to August 2020. A total of 150 references were included in the final qualitative analysis.

## Sexual Dimorphism and Anti-Infectious Immunity

Most epidemiological studies have shown that being a man is a risk factor for infectious diseases. Hence, women exhibit a higher ability to recognize pathogens, recruit more innate immune cells, and mount stronger adaptive immune responses than men. As a large body of literature has addressed the role of sexual dimorphism in anti-infectious immunity (5), our aim was to provide a summary of critical immune issues.

The innate recognition of pathogens involves numerous membrane and cytosolic molecules, including Toll-like receptors (TLRs). Whereas TLR4 expression is higher in neutrophils and macrophages from males, that of TLR7 is higher in females; this may be related to the hypothesis that X chromosome-encoded TLR7 can escape X inactivation, resulting in higher levels of TLR7 expression in females (5, 6). Notably, X- inactivation escape is also responsible for the female bias in autoimmune diseases (7). Furthermore, sexual dimorphism may affect the regulatory pathway of TLR7. Hence, the CD200 receptor, which is the receptor of the OX-2 glycoprotein, controls TLR7 responses. In its absence, type I interferon (IFN) production and viral clearance are increased in female mice infected with hepatitis coronavirus (8). Also, the lower expression of TLR9 in female mice infected with cytomegalovirus (CMV) likely accounts for the higher susceptibility and lower activation of their immune system (9).

The recruitment of innate immune cells is driven by inflammatory mediators. In response to lipopolysaccharide (LPS), circulating mononuclear cells from men produce more tumor necrosis factor (TNF) than those from women (10). This finding is consistent with the observation of sepsis, in which men produce higher inflammatory cytokines and lower anti-inflammatory cytokines than women (11, 12). On the other hand, mononuclear cells from men produce more IL-10, an immunoregulatory cytokine of innate and adaptive immune responses, in response to viral infections *via* TLR8 and TLR9 pathways, than mononuclear cells of women (13). It should be noted that the reaction to LPS can be influenced by the hypothalamic–pituitary–adrenal axis, in particular by the cortisol response to acute stress. Female mice were found to have a significantly higher concentration of cortisol when treated with LPS (14). In contrast, human immunodeficiency virus (HIV)-infected women showed blunted cortisol response to acute stress (15).

Moreover, adaptive immune responses seem more efficient in women than in men. The antibody response of women is usually higher than that of men (16). Women exhibit an increased number of immunoglobulin-producing B cells and higher baseline immunoglobulin levels. The repertoire of B cells has been reported to amplify this difference (17). This is illustrated by differences in antibody repertoire and avidity in men and women following H1N1 vaccination (18). Sexual differences have also been reported when it comes to cellular immune response. Women have higher baseline CD4^+^ T cell counts and higher CD4^+^/CD8^+^ ratios than age-matched men, whereas men have a higher baseline CD8^+^ T cell number (16). After *in vitro* stimulation of mononuclear cells, women have been shown to have higher numbers of activated and proliferating T cells (16). It has been established that human leukocytes antigen (HLA) molecules shape the repertoire of T cell receptors (TCRs), as shown by HLA-mediated use of TCR V beta chains. Their use differs in men and women and is likely under steroid control (19). Hence, the fight of the immune system against infectious pathogens relies on sexual dimorphism.

## Sex Hormones and Control of Infections

### Sex Hormones in Males and Nonpregnant Females

**Figure 2:**
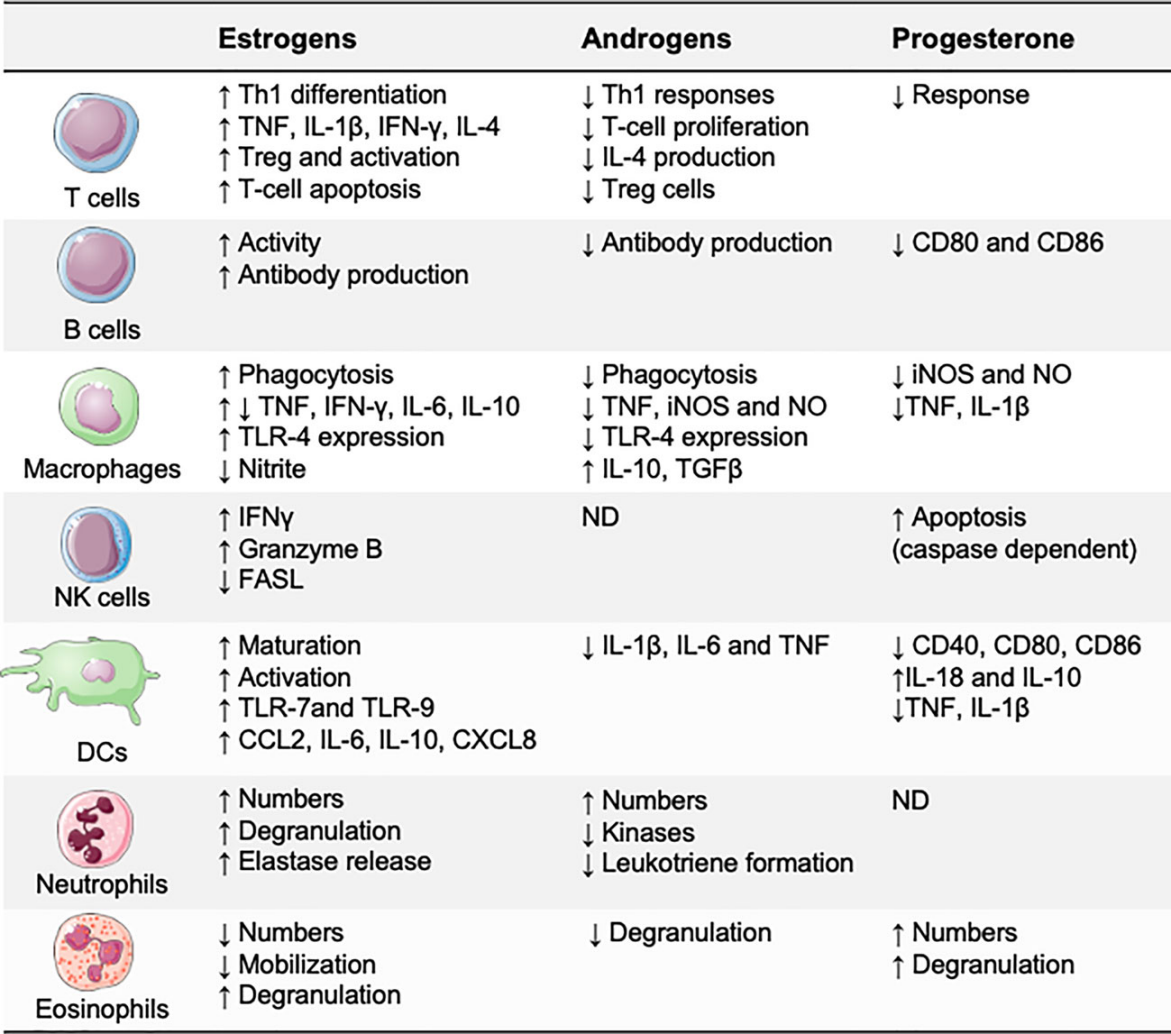
Effects of sex hormones on the immune system. Estrogens, progesterone, and androgens may directly affect immune cell functions. Generally, testosterone and progesterone are anti-inflammatory, suppressing several of the immune responses necessary for inflammation, whereas estradiol has bipotential effects: proinflammatory at low concentrations and anti-inflammatory at high concentrations. CCL, CC−chemokine ligand; CXCL, CXC-chemokine ligand; FASL, FAS ligand; iNOS, inducible nitric oxide synthase; ND, not defined; NO, nitric oxide; Treg, regulatory T.

Sex hormones modulate immune responses through specific receptors, which are expressed by most immune cells, including lymphocytes, macrophages, and dendritic cells (DCs) (20). After the hormone binds to its receptor, the hormone–receptor complex moves to the nucleus of the immune cell and binds to promoters that contain specific hormonal response elements (21). These hormonal response elements are present in the promoters of several innate immunity genes, suggesting that sex hormones may directly affect the functions of immune cells (**Figure 2**). Estradiol has probably been the most extensively investigated sex hormone. Low estradiol concentrations favor Th1-type responses and cell-mediated immunity, whereas high estradiol concentrations induce Th2-type responses and humoral immunity (22, 23). Progesterone and testosterone are known to have broad anti-inflammatory effects and to suppress innate immune responses (22, 24).

Sex hormones play an important role in susceptibility to infections, and tuberculosis is one of the most convincing examples, even though the evidence of such is based on unethical experiments. In 1969, research showed that the mortality rate of *Mycobacterium tuberculosis* infection was markedly lower in castrated men than intact men (25). In contrast, the tuberculosis-associated mortality rate in ovariectomized women is higher than in non-ovariectomized women (7.0% *vs.* 0.7%) (26). These two studies have shed light on the protective role of castration and the deleterious role of oophorectomy in humans, suggesting that testosterone may favor mycobacterial diseases, whereas estrogen may be protective. In a similar vein, postmenopausal women with low estrogen levels are more susceptible to opportunistic infections, as shown by an increased risk of pulmonary nontuberculous mycobacterial infections (27). It is noteworthy that, in a small group of HIV-infected women treated with hormone replacement therapy, a reduced risk of death was observed (28).

Clinical observations are not sufficient to understand the effects of sex hormones in susceptibility to infection, and only experimental models clearly depict the susceptibility and severity of infection as well as its pathophysiological mechanisms (**Table 1**). The castration of male mice reduces host sensitivity to certain infections, whereas the administration of testosterone into female mice increases their susceptibility to these infections (35, 40–43, 49). Estradiol protects female rats against bacterial sepsis and attenuates tissue lesions induced by *Helicobacter pylori* in mice (31, 32, 37). In *Coxiella burnetii* infection of mice, tissue infection and granulomatous responses are largely under the control of estrogens: the treatment of ovariectomized mice with 17β-estradiol reduces bacterial loads and granuloma numbers (29). We have clearly shown that the response to *C. burnetii* infection is sex dependent and that sex hormones are responsible for more than 60% of gene modulation in this specific infection (50). These modulated genes in infected females are involved in the circadian rhythm pathway, such as *Clock* (down-modulated) and *Per* (up-regulated), which interfere with the production of estradiol (51). In infected males, modulated genes are associated with an anti-inflammatory response, in particular, IL-10 overproduction, which is known to be associated with a progression toward persistent *C. burnetii* infection (52, 53).

**Table 1:** Effect of sex hormones on diseases in animal models.

Infections		Models	Susceptibility and severity	Hormonal effects	References
Bacterial	*C. burnetii*	C57BL/6 mice	M > F	ovariectomy: resistance lost	(29)
				oestradiol treatment after ovariectomy: resistance restored	
	*C. trachomatis*	Lewis rats	–	oestradiol treatment after ovariectomy: ↓susceptibility	(30)
				progesterone treatment after ovariectomy: ↑susceptibility	
	*E. faecalis (i.e. administration)*	Wistar rats	–	oestradiol treatment after ovariectomy: ↑protection against sepsis	(31)
	*H. pylori*	INS-GAS mice	M > F	oestradiol treatment: ↓severity of gastric lesions	(32)
	*L. monocytogenes*	C57BL/6 mice	F > M	oestradiol treatment: ↓resistance by inhibiting IL-2 production and subsequent T cell proliferation	(33)
	*M. avium*	DBA/2 mice	M > F	ovariectomy: ↑susceptibility	(34)
				oestradiol treatment: susceptibility mitigated	
	*M. marinum*	BALB/c mice	M > F	castration: ↓severity	(35)
	*M. intracellulare*				
	*M. tuberculosis*	BALB/c mice	M > F	testosterone treatment of female or castrated mice: ↑susceptibility	(36)
	*V. vulnificus LPS (intravenous injection)*	Sprague-Dawley rats	M > F	ovariectomy:	(37)
				↑mortality similar to males	
				oestradiol treatment after gonadectomy of males and females: ameliorated the severity of disease	
Viral	Herpes simplex virus (HSV)-2	C57BL/6 mice	–	progesterone treatment in females: ↑susceptibility	(38)
				↓protective immune response	
				oestradiol treatment: ↑protection	
	Simian immunodeficiency virus (SIV)	Macaques	–	progesterone treatment: enhanced SIV vaginal transmission and disease course oestradiol treatment: protect against vaginal transmission of SIV	(39)
Parasitic	*E. histolytica*	C57BL/6 mice	M > F	testosterone treatment in females: ↑severity	(40, 41)
		Syrian hamsters		castration: ↓severity	
				gonadectomy: inhibition of amoebic liver abscess development	
	*Leishmania* spp.	Syrian hamsters	M > F	testosterone treatment in females: ↓severity	(42)
	*Plasmodium* spp.	C57BL/6 mice	M > F	testosterone treatment of females: ↑severity and mortality	(43)
				castration: resistance	
	*S. mansoni*	CBA/J mice	F > M	oestradiol treatment of males: ↑parasite load	(44)
	*T. crassiceps*	BALB/c mice	F > M	castration: ↑number of parasites	(45)
				ovariectomy: ↓number of parasites	
	*T. gondii*	C57BL/6 mice	F > M	testosterone treatment in females: ↓severity and mortality	(46)
				oestradiol treatment of males: no effect	
				castration: dissolves sex bias	
Fungal	*C. albicans*	CFW mice	more rapid clearance in females	gonadectomy: lower initial incidence of infection in females, but not in males in both sexes: ↑clearance of candiduria	(47)
	*P. brasiliensis*	C57BL/6 mice	M > F	oestradiol treatment after castration: ↓IL-10, ↓severity testosterone treatment after ovariectomy: ↑IL-10	(48)
			↗ clearance in females		

Susceptibility to infection and the effects of sex hormones are described by comparing males (M) and females (F). The term ovariectomy is used only in the case of females, the term castration only for males and the term gonadectomy when it is used on males and females.

Some pathogens have evolved mechanisms that can manipulate host sex hormone production (21). *Schistosoma haematobium* produces an estrogenic compound that antagonizes both the signaling and expression of estrogen receptors, favoring its replication (54). Similarly, *Taenia crassiceps* enzymatically reduces testosterone concentrations and increases estradiol concentrations, and these effects promote the reproduction of parasites in male rodents (55). Androgen response elements have been identified in hepatitis B virus (HBV), allowing testosterone to affect HBV replication by direct binding to these elements (56). Interestingly, the genome of human papillomavirus (HPV) high-risk types 16 and 18 contains a progesterone response pattern that can stimulate HPV replication, playing an important role in the HPV-induced transformation process (57). Thus, increased progesterone concentrations may explain the higher frequency of malignant HPV lesions in women than in men. Progesterone inhibits the transition of *Candida albicans* into a hyphal form, whereas estradiol stimulates this transition, increasing fungal virulence (58). In fact, an important corpus of data clearly indicates that sex hormones play a major role in host defense and microorganism lifecycles.

### Sex Hormones in Pregnant Women

Pregnancy is a period of major hormonal change with a peculiar susceptibility to infections and a risk of complications for the fetus and mother. During the first trimester, the risk of transmitting infections to the fetus is rare but often results in miscarriage, while infections in the third trimester can result in maternofetal complications (**Table 2**) (3, 71). Susceptibility to listeriosis, toxoplasmosis, candidiasis, and HIV infection is known to increase during pregnancy (72). The risk of contracting listeriosis is 20 times higher in pregnant women than in nonpregnant women, and the risk of toxoplasmosis seroconversion increases by 2.2 times during pregnancy (3).

**Table 2:** Infections that cause adverse pregnancy or foetal outcomes.

Infections		Most at-risk trimester	Maternal clinical manifestations and severity	Maternal risk of mortality	Foetal risk	References
Bacterial	*B. melitensis*	1st trimester	no specific clinical sign	yes	spontaneous abortion	(59)
					preterm birth	
					congenital brucellosis	
					mortality	
	*C. burnetii*	2–3rd trimester	higher risk of persistent *C. burnetii* infection	yes	intrauterine growth restriction	(60, 61)
					spontaneous abortion	
					preterm birth	
					foetal demise	
	*L. monocytogenes*	3rd trimester	sepsis, meningitis, rhombencephalitis	unknown	spontaneous abortion	(3, 62)
					preterm birth	
					serious neonatal disease	
					foetal demise	
	*C. trachomatis*	all	no specific clinical sign	unknown	spontaneous abortion	(63)
					preterm birth	
					premature rupture of membranes	
					low birth weight	
	Group B *Streptococcus*	delivery	bacteraemia, sepsis pyelonephritis,	unknown	preterm birth	(64)
					neonatal infection: sepsis, meningitis, pneumonia	
Viral	Zika	1st trimester	no specific clinical sign	unknown	microcephaly	(65, 66)
					ocular abnormalities	
					foetal demise	
	Parvovirus B19	1st and 2nd trimesters	acute arthritis and arthralgias	unknown	spontaneous abortion	(67)
					foetal complications (severe anaemia, hydrops fetalis)	
	Hepatitis B	3rd trimester	no specific clinical sign	unknown	low birth weight	(68)
					preterm birth	
					perinatal transmission	
	Hepatitis E	3rd trimester	fulminant hepatic failure	yes	preterm birth	(69)
					mortality	
	Herpes simplex virus	3rd trimester	no specific clinical sign	unknown	spontaneous abortion	(69)
					intrauterine growth restriction	
					congenital and neonatal herpes infections	
	Influenza virus	3rd trimester	severe disease, pneumonia, cardiopulmonary event	yes	spontaneous abortion	(70)
					preterm birth	
					mortality	
	Measles virus	3rd trimester	severe disease, respiratory complications, pneumonia, encephalitis	yes	spontaneous abortion	(3)
					preterm birth	
					congenital defects	
	Varicella virus	3rd trimester	severe disease, pneumonia	yes	mortality	(69)
					congenital varicella syndrome	
	CMV	all	no specific clinical sign	unknown	intrauterine growth restriction	(69)
					congenital infection	
					mortality	
	Ebola virus	all	severe bleeding	yes	spontaneous abortion	(69)
					preterm birth	
Parasitic	*Plasmodium* spp.	first half of pregnancy	severe anaemia, renal failure, higher frequency of lymphadenopathy	yes	low birth weight	(3)
					intrauterine growth restriction	
					preterm birth	
	*T. gondii*	1st trimester	no specific clinical sign	unknown	congenital diseases (microcephaly, intracranial calcifications)	(3)

Infections that can cause adverse pregnancy or foetal outcomes and are described according to the stage of pregnancy.

**Figure 3:**
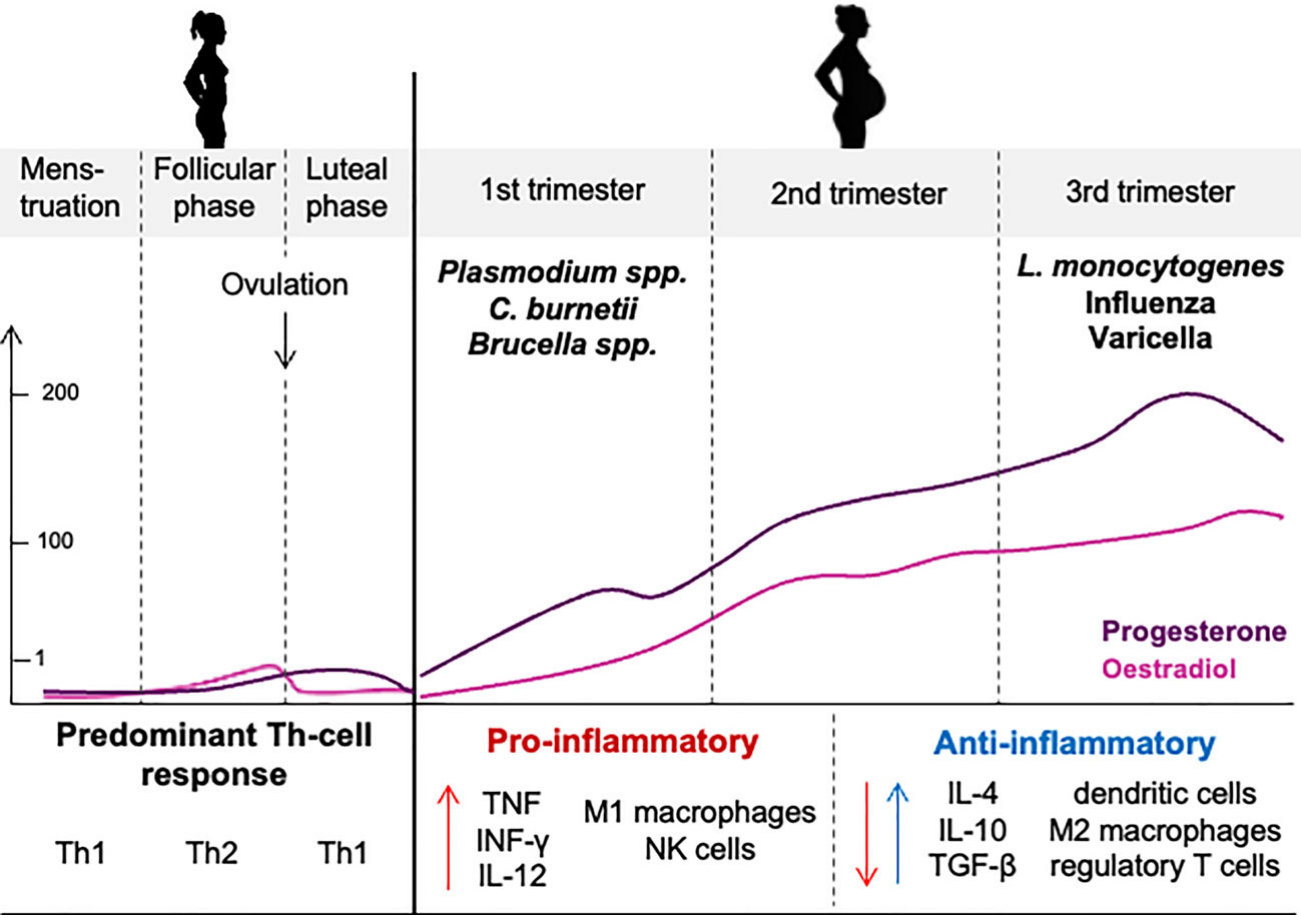
Hormone levels and immune responses during the menstrual cycle and pregnancy. Increased hormonal concentrations during pregnancy contribute to the immune shifts to support a successful pregnancy but also increase the susceptibility of women to infectious disease.

Infection severity is increased in pregnant women; they are seven times more likely to be hospitalized, twice more likely to die from an influenza virus, and three times more likely to develop severe dengue and lethal *Plasmodium falciparum* infections than nonpregnant women (3, 73–75). Pregnancy complications have been identified in more than 50% of pregnant women infected with *C. burnetii* infection (4). They include spontaneous abortion (26%), premature delivery (45%), or intrauterine growth restriction (5.3%) (4, 60, 61). Although women likely seem to be protected from infectious risk *via* estrogens, clinical observations suggest that, during pregnancy, high concentrations of estrogens are detrimental for the host response to infection (**Figure 3**).

During the third trimester, high concentrations of estradiol and progesterone prevent efficient Th1 immune responses and promote immunoregulatory Th2 immune responses, which is essential for the success of the fetal graft (76, 77). Unfortunately, the expansion of Th2 cells, which are known to decrease the robustness of protective cell-mediated immunity, is also, at least partially, responsible for altered immune responses to infections, which may account for the prevalence and severity of infections during pregnancy (78, 79).

## Genetic Factors and Sexual Dimorphism

Although the Y-chromosome, which is found only in men, encodes about 100 genes, 1,100 genes are expressed on the X-chromosome, which is shared by both men and women, including a large number of genes related to innate and adaptive immune systems. Among them there are genes encoding pattern recognition receptors (such as TLR7 and TLR8) (80) or transcriptional effectors (NF-κB) (81). To prevent excessive X-chromosome responses in women, one of the two X-chromosomes is inactivated. However, 15% of the X genes escape inactivation, and their copy number is higher in women than in men, which accounts for the increased risk of autoimmunity in women (6, 82). Similarly, the sex chromosomes show an unbalanced microRNA (miRNA) repartition. Indeed, the X-chromosome contains 113 miRNAs (10% of all miRNAs), whereas only two miRNAs are found in the Y-chromosome (83). These miRNAs, which are involved in the response to infection, are critical regulators of the immune response (84). As an example, mRNA-223 controls susceptibility to *M. tuberculosis* infection by regulating lung neutrophil recruitment (85). The incomplete inactivation of the X-chromosome leads to a higher expression of miRNAs in women. These differences attributable to X-chromosome inactivation contribute to sex differences in immune responses and, therefore, in susceptibility to infections. However, murine models could be considered biased for sex hormone analysis. The four core genotype mouse model enables the study of differences in gene expression solely due to sex chromosome complement without involvement of sex hormones (86). Such an approach is useful to assess the role of IL-1 receptor-associated kinase-1 (IRAK1), CD40 ligand *(CD40LG*), C-X-C motif chemokine receptor 3, and *IL13RA1* or *TLR7* genes known to escape the X-related inactivation process in infectious diseases (6, 87–89). These findings highlight enhanced female immune response to infectious agents through the involvement of X-chromosome-linked genes.

Chromosome polymorphism affects the severity of infections. Indeed, a single nucleotide polymorphism in the X-chromosome is associated with slow progression to AIDS in women but not in men (90). TLR8 gene polymorphisms on X chromosomes are related to susceptibility to tuberculosis, particularly in boys (91, 92). Other genetic variations independent of sex chromosomes participate in sexual dimorphism, including IL-6 promoter polymorphisms associated with the development of chronic hepatitis C virus (HCV) infection, which is primarily reported in men (93). In contrast, different polymorphisms in the gene encoding CTLA-4 associated with HCV infection recovery are more frequent in women (94). In the context of COVID-19, it has been shown that the ACE-2 receptor, known to recognize SARS-CoV2, is encoded by the X chromosome, which may partly explain the sexual dimorphism of COVID-19 (95).

Genetic variations in the Y-chromosome increase mouse susceptibility to influenza A virus infection (96) and have a direct effect on the survival of coxsackievirus B3-infected mice (97). Genes encoding resistance to *Plasmodium chabaudi*, *Leishmania mexicana*, or mousepox infection have been identified in mice on autosomal chromosomes (**Table 3**), and these loci confer greater resistance to females (98–100). Thus, genetic resistance to infection is partly sex dependent, but this association requires further investigation.

**Table 3:** Genetic susceptibility to infections in mice.

Infections	Genetic variation	Effect of genetic variation	References
Influenza A virus	chromosome Y	↗ susceptibility to infection of males	(96)
		↗ pathogenic immune responses in lungs	
Coxsackievirus B3	chromosome Y	↗ mortality in infected males	(97)
	**Resistance genes**	**Effect of resistance genes**	
*P. chabaudi*	Char 1-4	greater resistance of females (Char 2, Char 4)	(98)
*L. mexicana*	Scl-2	greater resistance of females	(99)
Mousepox	Rmp1–4	greater resistance of females (Rmp 2, Rmp 4)	(100)

Genetic variations in the Y chromosome influence the survival of male mice following influenza A virus and coxsackievirus B3 infection. Resistance genes have been identified, and the effects of these genes confer greater resistance to infections for female mice.

## Sexual Dimorphism in Infectious Diseases: Clinical Evidence

Epidemiological studies and clinical observations provide clear evidence of sexual bias in infectious diseases and offer insights into exploring the role of sexual dimorphism in infectious diseases. These studies refer to the differences of susceptibility and clinical presentation in men and women according to the site of infection or the nature of the microorganism.

### Sexual Dimorphism Related to the Site of Infection

**Figure 4:**
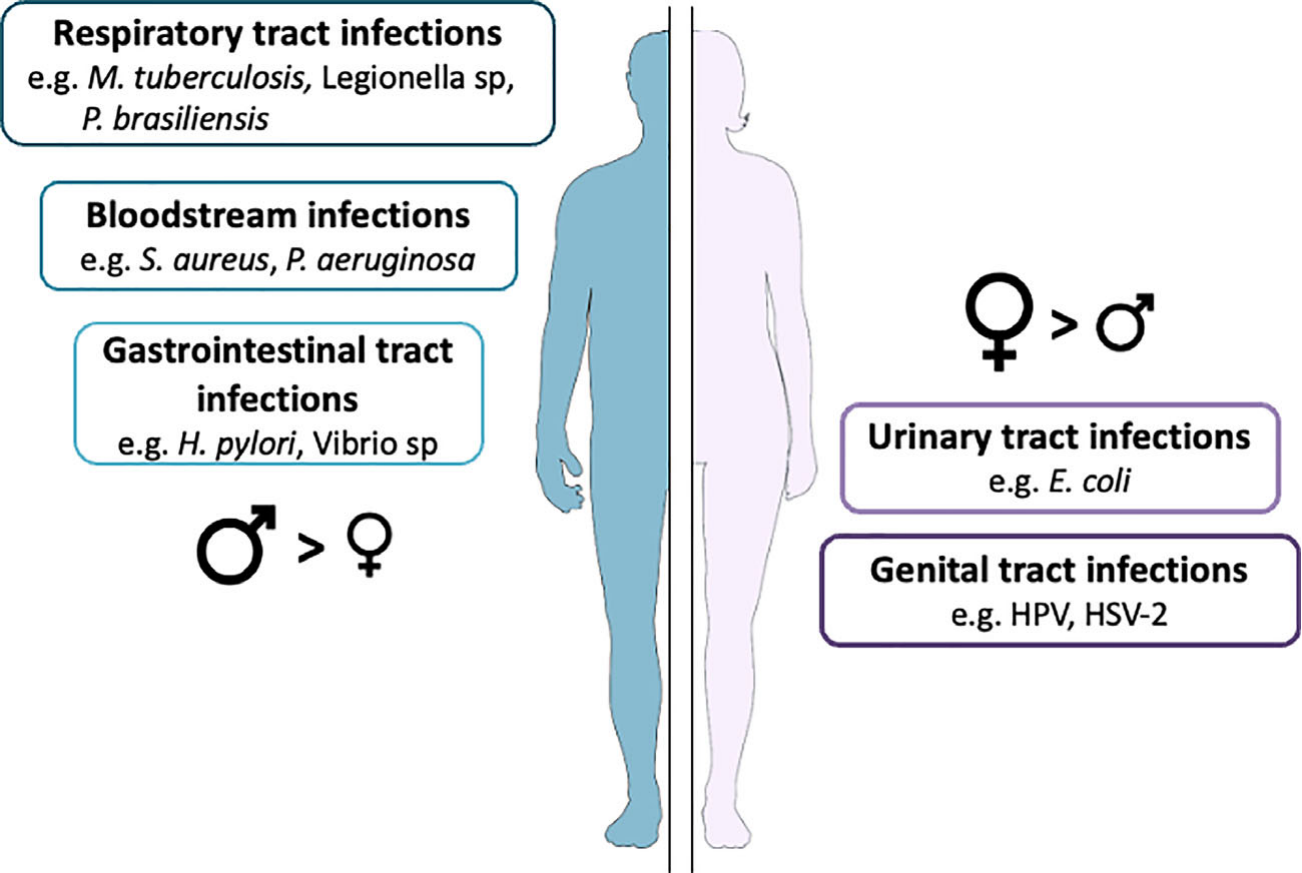
Infection prevalence according to anatomical characteristics. Data extracted from a long-term cohort followed in our institution.

The incidence of urinary tract infections, genital infections, infective endocarditis, and respiratory tract infections differs between men and women. We have summarized the main findings about sexual dimorphism in infections in which anatomical differences might play a role in **Figure 4**.

Urinary tract infections are the most frequent infections worldwide, occurring in 53,067 cases per 100,000 women and 13,689 cases per 100,000 men. Women from the ages of 16 to 35 are about 35 times more likely to develop urinary tract infections than men (101). Although anatomical differences have long been proposed as the main explanation for the predominance of women experiencing more urinary tract infections, other factors are emerging that more fully explain the increased risk of urinary tract infections in women; these factors include sexual intercourse, antimicrobial exposure, estrogenic status, contraception influencing the vaginal microbiota, and any intervention resulting in the loss of normally protective *Lactobacillus* spp (102, 103). The distribution of the causative agents of urinary tract infections also differs in men and women: *Escherichia coli*, *Klebsiella pneumonia*, and *Streptococcus agalactiae* are more frequently found in women, whereas *Enterococcus faecalis*, *Proteus mirabilis*, and *Pseudomonas aeruginosa* are more often found in men (104). Cellular factors also differ according to sex. Hence, the epithelial cells of the penile skin appear to be more resistant to HPV infection than the cervical epithelium, the former being keratinized and the latter mucosal (105).

Infective endocarditis exhibits a male/female (M/F) sex ratio above two (106). The site of infection, which is the injured valve, is more often the mitral valves in women (50% *vs.* 36%, *p* = 0.02) and the aortic valves in men (46% *vs.* 31%, *p* = 0.02) (106). Infective endocarditis-associated in-hospital death is higher in women than in men. Nevertheless, it is difficult to establish an association between sex and both the site of infection and increased risk of infective endocarditis, because age and comorbidities such as diabetes mellitus are likely involved (106, 107).

Men develop lower respiratory tract infections more frequently than women, as shown in a two-year European prospective study evaluating the incidence of community-acquired pneumonia (16 *vs.* 9 cases per 10,000 person-years) (108, 109). In contrast, sinusitis and tonsillitis are more frequent in women than in men; here, the smaller size of paranasal sinus ostia in women may account for such dimorphism (110).

### Sexual Dimorphism Related to Microorganisms

In general, men are more affected by bacterial and parasitic infections than women, with the exception of *Escherichia coli*, *Borrelia burgdorferi* and *Chlamydia trachomatis*. In this section, we focus on infectious diseases for which a pronounced sexual dimorphism has been reported at clinical and epidemiological levels.

#### Bacterial Infections

**Figure 5:**
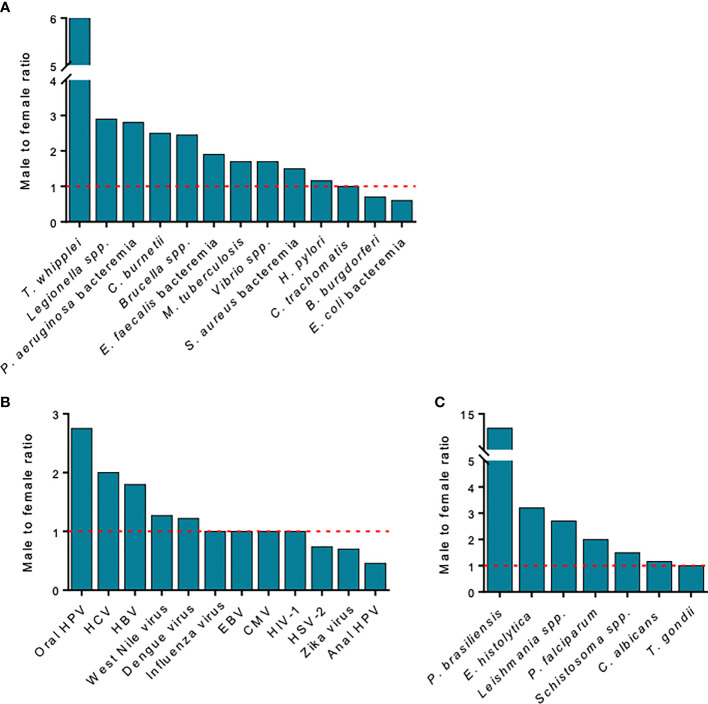
Sex differences in the prevalence of infections. The male-to-female (M/F) ratios for different **(A)** bacterial, **(B)** parasitic and fungal, and **(C)** viral infections are presented. *T. whipplei* infection occurs in six men for every one woman, but prevalence is similar between men and women for *Toxoplasma gondii* infection, whereas anal HPV infections are more frequent in women. Data extracted from (111).

Numerous infections caused by intracellular pathogens, such as *M. tuberculosis*, *C. burnetii*, *Legionella* sp., and *Brucella* sp., exhibit unbalanced M/F ratios (**Figure 5A**). We illustrate this point with three examples. Tuberculosis (defined by a clinical diagnosis of tuberculosis disease) exhibits a global M/F ratio of 1.7 (112). Although it is estimated that one-third of the world’s population has latent *M. tuberculosis* infections, it is also estimated that asymptomatic carriage affects more men than women (33% *vs.* 25%) (113). Among both HIV-negative and HIV-positive individuals, tuberculosis-related mortality is higher in men than in women, with an M/F ratio of 1.8 to 1.9, which is partly related to differences in gender access to healthcare (112). Interestingly, women more often develop extrapulmonary forms of the disease, with M/F ratios ranging from 1:1.7 to 1:2.9 in low-income countries (114).

**Figure 6:**
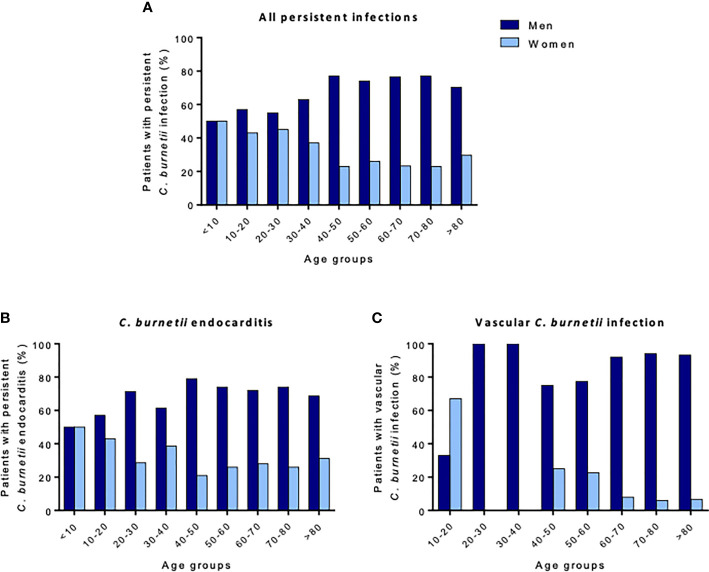
Percentage of patients with persistent *C. burnetii* infection regarding sex and age. The proportion of men and women patients for **(A)** all persistent *C. burnetii* infections, **(B)** *C. burnetii* endocarditis, and **(C)** vascular *C. burnetii* infections are presented. In children, persistent *C. burnetii* infection affects girls and boys similarly, whereas adult men are mostly affected. After 40 years of age, men represent more than 70% of patients.

From the French National Reference Centre for Q fever, the sexual dimorphism was more pronounced during *C. burnetii* persistent focal infection (M/F sex ratio: 2.8) than during acute Q fever (M/F sex ratio: 2.2). The M/F sex ratio reached 7.5 in patients with vascular *C. burnetii* infection, independent of age, whereas the sex ratio related to mortality was 3.8 (4, 115). These differences were not found in children, in whom boys represented 47% of the cohort (**Figure 6**) (4). In addition, being male was associated with a higher risk of exhibiting G-isotype anticardiolipin antibodies, which are associated with acute Q fever complications (*OR* 1.6 [1.3–2.1], *p* < 0.001) (116).

*Tropheryma whipplei* is a bacterium that causes a rare, chronic, and systemic disease: Whipple’s disease. The classical presentation of the disease is characterized by malabsorption syndrome and arthralgias (117). The M/F ratio is 6.0 (118). In rural communities in Gabon, the prevalence of *T. whipplei* is 23% in men and 16% in women (*p* = 0.05), and being a man is identified as a risk factor for *T. whipplei* asymptomatic carriage (*OR* 1.6, *p* < 0.05) (119).

**Figure 7:**
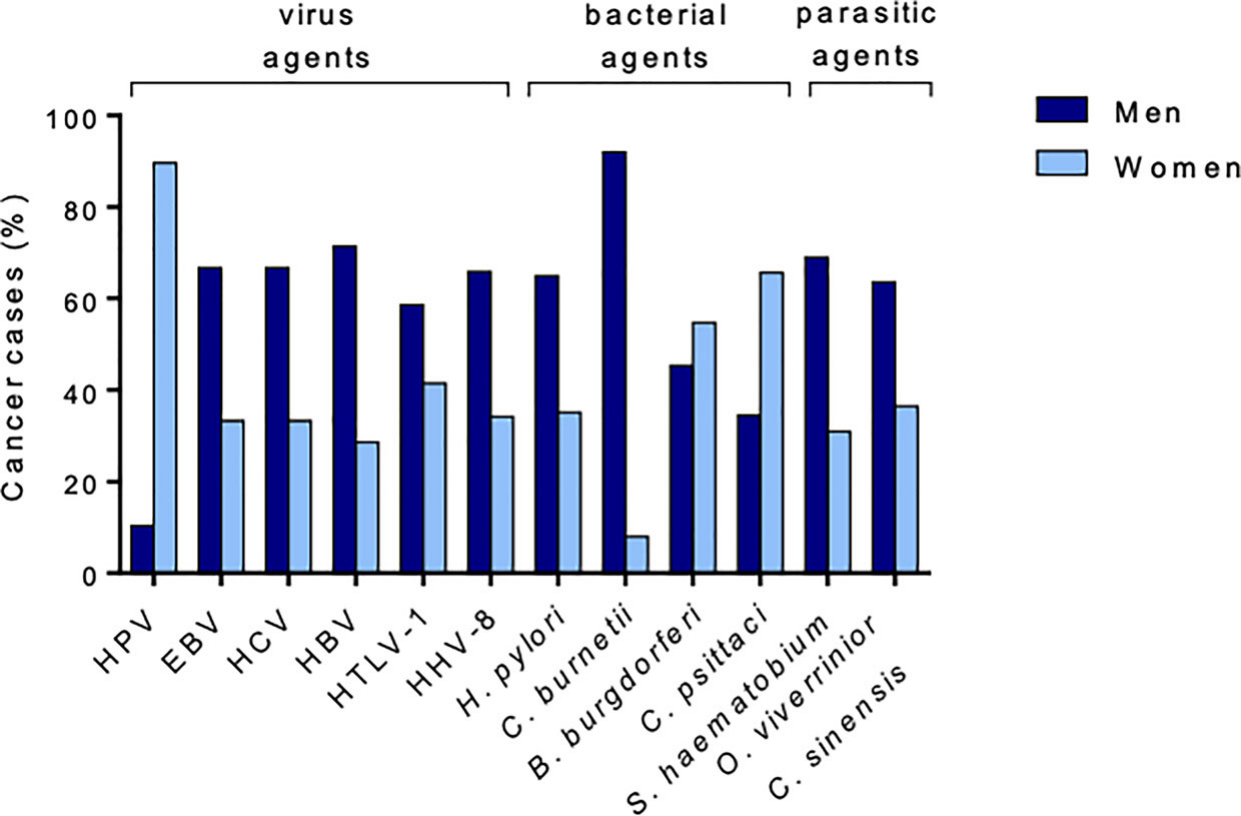
Percentage of cancer cases attributable to infectious agents by sex. Sex disparities are observed in infection-associated cancers. Globally, men are more likely to develop cancer because of infection, except in a few cases, such as HPV infection.

Sexual dimorphism is also found for cancers associated with bacterial infections (**Figure 7**) (120–125). Compared with women, men have a higher incidence of gastric adenocarcinoma associated with *H. pylori* infection and non-Hodgkin lymphomas associated with *C. burnetii* infection than women (122, 123). In contrast, women are more susceptible than men to developing ocular adnexal lymphoma associated with *Chlamydia psittaci* infection and primary cutaneous B-cell lymphomas associated with *Borrelia burgdorferi* infection (124, 125).

Overall, bacterial infections and their consequences, such as infection-associated cancers, are more pronounced in men.

#### Parasitic and Fungal Infections

Sexual differences are particularly pronounced in parasitic infections (**Figure 5B**) (126–128). Although asymptomatic carriage of *Entamoeba histolytica* is similar in both sexes, invasive amoebiasis affects a higher proportion of men than women (126). Men are also more affected by *P. falciparum* and *Plasmodium vivax* infections than women, whereas the mortality associated with *P. falciparum* is higher in women than in men (18% *vs.* 7.6%). This observation could be partly explained by anemia along with immunological differences, which is more frequent in pregnant women than in nonpregnant women, the latter being associated with increased mortality in *P. falciparum* infection (127).

There are sex differences in the incidence or manifestations of fungal infections. In paracoccidioidomycosis, this is caused by the fungus *Paracoccidioides brasiliensis*, where the M/F ratio is extremely high (**Figure 5B**) (128). As reported for bacterium-associated cancer, parasite-associated cancer exhibits sexual dimorphism. Men account for more than two-thirds of the cases of cholangiocarcinoma associated with *Opisthorchis viverrini* and *Clonorchis sinensis* and are more affected by urinary bladder cancer caused by *Schistosomia haematobium* (129, 130).

#### Viral Infections

Sexual dimorphism is classically less pronounced in viral infections, albeit differences between men and women have been reported (**Figure 5C**) (74, 131, 132). HBV and HCV infections are more frequent in men. Women spontaneously clear HCV more efficiently than men (45% *vs.* 34%) (131, 133). Finally, some disparities have been observed in infections caused by HIV-1. The HIV-1 viral load in untreated women is up to 40% lower than in men, but progression to acquired immunodeficiency syndrome (AIDS) is faster in women at similar viremia levels, partly because of increased immune activation in women.

Sex differences have been reported in the incidence and severity of dengue virus infection. The incidence of this infection is higher in men than in women (from 1.5:1 to 2.5:1) (134). In a report from Fiji, men were more likely to develop a lethal presentation than women (63% of deaths occurring in men) (135). These data could partly be explained by a higher occupational exposure in men, but also by a sex dimorphism in the antibody enhancement process. In fact, pre-existing sub-neutralizing levels of antibodies, through Fcγ receptor interaction, can lead to increased intracellular viral uptake and enhanced infection (136). This is illustrated by enhanced dengue severity mediated by maternally acquired heterotypic dengue antibodies (137). However, more animal and clinical studies are needed to explore sex differences in this phenomenon.

A sexual dimorphism has been identified in COVID-19 infections. Men were significantly more represented in the group of critical and deceased COVID-19 patients. Conversely, being a woman was associated with a reduced risk of death and a reduced risk of disease progression (138, 139). In fact, SARS-CoV-2 binds to ACE-2, a protein encoded by the genes of the X-chromosome (95), which is one of the hypotheses for explaining the sexual dimorphism observed in this disease. Recently, we identified an almost exclusively male population suffering from severe COVID-19 and characterized by the neutralization of type I IFN *via* inborn defects or the presence of antitype 1 IFN auto-antibodies (140). It has also been shown that post-acute COVID-19 syndrome is more likely to occur in women than in men.

Finally, there is sexual dimorphism in virus-associated cancers, as reported for cancers associated with bacteria or parasites (141–143). After viral hepatitis, men have a two- to four-fold increased risk of developing hepatocellular carcinoma (141). Similarly, after human T cell leukemia virus type 1 infection, men can be up to 3.5 times more susceptible to developing adult T cell leukemia than women (142, 143).

## Sexual Dimorphism From a Gender Perspective

Although hormonal and chromosomic hypotheses are tempting to consider, they cannot explain all the features of sexual dimorphism in infectious diseases. Because of the intricacy of social and biological mechanisms in sex-associated susceptibility to infections, it is necessary to evaluate the influence of sociocultural factors, both at the contextual and individual levels. The latter are critical for analyzing the biological studies in which they may be confounded.

The historical and social organization that is partly based on gender roles may put men and women at risk of contracting specific infections in different ways. Mine workers are five to six times more likely to contract tuberculosis than the general population (144). People working in sewage treatment are exposed to *T. whipplei*; dockworkers, fishermen, or oyster shuckers to *Vibrio* sp. infection; slaughterhouse workers to Q fever; and farmers to brucellosis (145, 146). Slaughterhouse workers and farmers are predominantly male in developing countries (67%), while in industrialized countries, men and women are equally represented in these occupations (147). In the case of schistosomiasis, boys are infected more often than girls (ratio at 1.8), a difference persisting in adults (ratio at 1.5). This is partly explained by the more frequent bathing of boys in stagnant waters (148–150). There is no occupational exposure to *C. burnetii* (4, 151) at variance with brucellosis and bilharziosis (150, 152, 153).

In sex workers, sexually transmitted infections are more frequent in women than in men, and women sex workers are 13.5 times more likely to contract HIV than other women aged 15–49 years old, even in countries with a high HIV prevalence (154, 155). In developed countries, *Treponema pallidum* infection affects men in 88% to 96% of cases, most of them being men who have sex with men (58% to 82%) (156).

Moreover, at the individual level, health behaviors such as healthy lifestyle habits or healthcare-seeking behaviors could explain the gap in vulnerability that can be observed between women and men in terms of specific infectious diseases.

Comorbidities and lifestyle habits influence susceptibility to infections. Consumption of alcohol and tobacco increases the risk of infections, and their complications are higher in men than in women (157). Alcohol consumption is a risk factor for tuberculosis and accelerates liver fibrosis in HCV-infected individuals (158). Smoking is a risk factor for infectious lung diseases, accounting for up to 30% of the variance in the sex ratio of pulmonary tuberculosis notifications (159). In South India, the risk of progression toward pulmonary tuberculosis shows an M/F ratio of 2.7, whereas the exclusion of smokers and alcohol users reduces the M/F ratio to 1.2 (160). Also, high blood pressure and diabetes mellitus are more common in men than women (161, 162).

Finally, gender disparities in the use of healthcare services may influence the course of infectious diseases. For tuberculosis, prevalence surveys show that men are underdiagnosed as compared with women, suggesting that men may be less likely to seek or access healthcare than women (112, 163). Consequently, male tuberculosis cases may be underestimated in some countries (112, 163). Similarly, antiretroviral therapy coverage among people living with HIV is higher among women (52%) than men (41%) (132). This gender gap is likely related to gender norms. Among adult men, the delayed initiation of treatment and reduced adherence to treatment contribute to an increased number of deaths (132).

## Conclusion

Infections are multifactorial diseases whose exposition and evolution are influenced by social organization and health behaviors. Biological determinants also influence the course of infectious diseases, which are partially dictated by immune response and subjected to hormonal influences and chromosomal predispositions. Thus, susceptibility to infections changes over the course of an individual’s life. After puberty, women are initially less susceptible to infectious diseases because of their ability to mobilize and activate innate and adaptive immune responses, whereas during pregnancy, an immunoregulatory Th2 immune response is promoted. In men, testosterone has immunosuppressive properties. In addition, genetic factors determine the outcome of infections and are involved in susceptibility and resistance to microbial agents. Some microorganisms have developed strategies that take advantage of hormonal or chromosomal influences to survive and multiply. Therefore, sexual dimorphism needs to be integrated into the development of anti-infectious treatments.

## Author Contributions

Data curation: LG, CM, IL. Supervision: J-LM, ML. Writing – original draft: LG, CM, IL, M-KB. Writing – review and editing: SM, CD, DR, J-LM, ML. All authors contributed to the article and approved the submitted version.

## Conflict of Interest

ML served as speaker for MSD, Aspen and as consultant for Amomed, Ambuand Gilead.

The remaining authors declare that the research was conducted in the absence of any commercial or financial relationships that could be construed as a potential conflict of interest.
